# Facilitators and barriers for successful retirement: a qualitative study

**DOI:** 10.11604/pamj.2023.44.111.35608

**Published:** 2023-02-28

**Authors:** Zohreh Alavi, Yadollah Abolfathi Momtaz, Fardin Alipour

**Affiliations:** 1 Department of Social Work, School of Behavioral Sciences, University of Social Welfare and Rehabilitation Sciences, Tehran, Iran,; 2 Iranian Research Center on Aging, University of Social Welfare and Rehabilitation Sciences, Tehran, Iran,; 3 Malaysian Research Institute on Ageing, University Putra Malaysia, Serdang, Malaysia

**Keywords:** Barriers, facilitators, retirement, retirement adaptation, successful retirement

## Abstract

**Introduction:**

a retirement is a major event and a life-changing transition in human life. As retirement is accompanied by new needs and roles, there is a need for adaptation. This study intended to identify facilitators and barriers to a successful retirement.

**Methods:**

this qualitative study was conducted on 22 retirees and professionals in the field of retirement. The participants included 13 retirees (9 males and 4 females), 4 about-to-retirement individuals (3 females and 1 male), and 5 experts in the field of gerontology, retirement, and social sciences. The participants were selected using the purposive sampling technique. Data analysis was performed using conventional content analysis and Graneheim and Lundman´s methods.

**Results:**

in this study mean age of participants in the retiree group was 63.15±6.30 years. The mean age for the About-to-retirement group was 53.75*2.63 years, and their mean job experience was 29 years. Also, the expert group had an average of 15 years of experience in their field. Facilitators were classified as benefiting from social support systems, personal characteristics, and social participation. Barriers were categorized into four groups, namely worsened health status, lack of a plan and retirement crisis, socioeconomic problems, and inadequate support systems.

**Conclusion:**

this study considered retirement not only as a phenomenon accompanied by several psychosocial challenges but also as an opportunity for growth that various factors either facilitate or hinder. In order to promote current services, it would be helpful to consider accompanying changes and the facilitating or hindering factors of retirement adaptation. Therefore, future retirement preparation programs should consider these factors.

## Introduction

Projections about population growth and life expectancy in Iran are similar to other countries [1-3]. According to recent projections, Iran´s population will grow by 30% by 2050 [4], in which retirees account for a substantial portion of the population. That is, previously active workers will account for a large portion of the population. There are various definitions for retirement, including the time of life when one chooses to permanently leave the workforce, leaving the formal workforce market [5], reducing working hours during the career (e.g., less than full-time), receiving social security benefits or defining and recognizing him/herself as a retiree [6,7]. The Iranian standard of working time is considered to be between 25 and 35 years, and the age of retirement is determined by law, starting around 60. Retirement years can differ a little based on a few factors such as job characteristics (such as profession and hardship in the workplace) or gender [8].

Retirement, as a life-changing transition in human life, is a major event and transition [9], and due to new conditions, desires, and roles, it requires adaptation. Therefore, adjustment to a successful retirement is essential for maintaining physical and mental well-being during this period. A wide range of meanings accompanies retirement. Exiting from work, losing financial and social job-related factors, and separation from time structures and work responsibilities affect changing individuals´ lifestyles [5,10,11]. Over time, changes in the concept of retirement have transformed from the traditional definition of an event at a legal age to an active change in life [12,13] that is accompanied by a transition. In addition, a successful retirement is described in various aspects, indicating the necessity of investigating a successful retirement from financial, social, and psychological features [14,15].

Studies demonstrated that retirees might experience disturbances in social interaction and participation and decreased social dignity or isolation [16,17]. Previous studies reported low life expectancy, loneliness, anxiety [18,19], increased stress resulting in psychosomatic diseases [20], shock and disbelief, emptiness and helplessness [21], depression [22], decreased general health [23], and reduced problem-solving ability and feeling of control [24] as some major events of retirement. Such issues will translate into declined quality of life and even an increased chance of death [25]. Role theorists argue that the loss of functional work-related roles is remarkable, so retirement transition can cause people to feel psychological issues, leading to low levels of well-being [7]. Therefore, policymakers require evidence and plan to better manage the processes and consequences of retirement [17,26] to achieve a successful retirement.

However, most studies focused on the economic aspects of retirement [27]. A successful retirement can be defined as the ability to adapt to a new situation that as leaving work, or forgetting a previous role while facing physical or psychological consequences of retirement(e.g., declined scope of physical activity, low physical health, and declined life satisfaction and happiness), and successful retirement perception. In other words, a successful retirement requires flexibility and adaptation to available resources (i.e., wide gains and few absences) [28]. Failure in maintaining the standard of living, in comparison to that before retirement, causes retirees to feel powerless, self-blame, and have low self-esteem [14]. Adaptation to retirement can be complex and might reflect different time patterns following retirement, which depend on different factors.

Retirement is part of life´s process that involves gains and losses, in accordance with lifespan theories. Due to limited resources, the retiree has to select important goals, develop strategies (e.g., information, skills) to cope with developmental challenges, and use alternative strategies [29]. So the identification of factors that affect the adaptation to retirement is necessary because weak and unsuccessful adaptation results in dissatisfaction, psychological problems, and physical problems [30]. Despite considering retirement a stressful event, not necessarily any retiree experiences negative experiences, and some can adapt to it [31]. As a result, retirement adaptation can improve health and quality of life [32]. Therefore, the identification of factors that either facilitate or hinder a successful retirement, as the purpose of this study, can be in line with interventions and policies intended for retirement adaptation [33].

## Methods

As this study intended to identify factors that facilitate or hinder a successful retirement, a qualitative study design and Graneheim and Lundman´s data analysis method were used [34]. This method is appropriate to describe a phenomenon with limited evidence. It is based on data immersion to achieve deep understanding without considering previous themes. Participants were selected from three groups, including retirees who had already retired, individuals about to retire, and experts. They had rich lived experiences and knowledge from different backgrounds. The purposive sampling technique was used while emphasizing variety. They were selected through personal contact, and an advertisement then explained to them by telephone or face-to-face methods Inclusion criteria (i.e., retirement duration of at least one year, about to retirement (less than one year), and expertise in the field of gerontology, retirement, and social science) have been followed in this study. Also, those who were not willing to participate were excluded from the study.

Before entering the study, a comprehensive introduction to the study protocol was provided to all eligible participants. If they agreed, interviews were held by the first author (a Ph.D. student in social work with sufficient experience and interest in the study field. The interviews were carried out individually and held in the interviewees´ preferred place (e.g., home, office, and park) or by phone. In-depth and semi-structured interviews were used in this study. The sessions were audiotaped after obtaining the interviewees´ approval. The interviews lasted for 50-90 minutes. The interviews started with an initial conversation and open-ended questions. The interview guide was developed based on expert opinions and four pilot interviews. The following questions were used to explain the factors that either facilitate or hinder a successful retirement: what factors contribute to a successful retirement or hinder it. Who is a successful retiree and what are the characteristics?

A probing question was used to acquire in-depth information (i.e., Can you explain more). In addition to audio recording, field notes, including nonverbal reactions, were also recorded. The transcripts were typewritten word-for-word by the first author. All interviews were read repeatedly after transcription. All transcripts were analyzed before conducting the next interview. Data analysis was performed simultaneously with interviewing the participants. Thirty participants approached this study, but eight refused to participate. Finally, Data saturation was achieved after 22 interviews. Samples included 13 retired school teachers living in Mashhad, Iran, (9 males and 4 females) (Table 1), 4 about-to-retirement school teachers (3 females and 1 male) (Table 2), and 5 experts in the field of gerontology, retirement, and social science (Table 3). Data were collected from February 2019 to September 2019 in Mashhad and Tehran.

**Table 1:** demographic characteristics of retired participants

Code	Age (year)	Gender	Educational level	Marital status	Current employment status	Spouse’s employment status	Time since retirement (year)	Interview location
1	66	Male	MSc	Married	Volunteer	Retired	9	Retirement association center
2	54	Male	Bachelor’s degree	Married	Part-time employment	Employed	3	Consultation center (workplace)
3	60	Male	MSc	Married	Part-time employment	Employed	3	Consultation center (workplace)
4	56	Female	MSc	Married	Volunteer	Self-employed	6	Retirement association center
5	61	Male	Associate’s degree	Married	Full-time employment	Housewife	11	Retirement association center
6	68	Female	Associate’s degree	Married	Unemployed	Retired	15	Park
7	64	Female	Diploma	Deceased	Full-time employment	Unemployed (deceased)	12	Retirement association center
8	67	Male	Diploma	Married	Part-time employment	Housewife	12	Park
9	69	Female	Diploma	Married	Volunteer	Retired	24	House
10	63	Male	Bachelor’s degree	Married	Part-time employment	Retired	13	Park
11	75	Male	Bachelor’s degree	Married	Unemployed	Retired	20	Park
12	65	Male	Bachelor’s degree	Widowed	Part-time employment	Deceased	13	Consultation center (workplace)
13	53	Male	Bachelor’s degree	Married	Full-time employment	Housewife	3	School (workplace)

**Table 2:** demographic characteristics of about-to-retirement participants

Code	Age (year)	Gender	Educational level	Marital status	Spouse’s employment status	Employment experience (year)	Interview type
14	55	Female	MSc	Married	Employed	29	Telephone
15	50	Female	MSc	Married	Self-employed	30	Telephone
16	54	Female	Bachelor	Married	Retired	29	Telephone
17	56	Male	Bachelor	Married	Employed	27	Telephone

**Table 3:** demographic characteristics of experts participants

Code	Gender	Educational level	Expert field	Occupation type	Experience (year)	Interview location
18	Male	PhD	Gerontology	Educational and practical	10	Office
19	Male	PhD	Gerontology	Educational and practical	12	Office
20	Female	PhD	Social science	Educational	11	Office
21	Female	PhD	Social science	Educational	8	Office
22	Male	Bachelor’s degree	Retirement	Practical	20	Telephone

Data analysis was performed using Graneheim and Lundman´s approach (2004) [34]. The interview scripts were written directly, in an informal manner, after the interview, while considering field notes, using Microsoft Word software. Then, data analysis was carried out using constant comparison and inductive content analysis. The data were categorized into meaning units, condensed meaning units, subcategories, main categories, and themes. Coding was performed by summarizing meaning units to reduce the number of units while respecting the original content. Qualitative studies measure credibility instead of reliability. In this study, credibility was ensured through the immersion of data, long-term contact with participants, and necessary revisions [35,36].

This study is confirmed by the Ethics Committee of the University of Rehabilitation and Social Health Sciences, Tehran, Iran (042. 1397. REC. USWR). Therefore, ethical research codes, such as being anonymous, using data only for research purposes, and the participant's right to contribute, stop the interview or refuse to answer any question considered in the study. In addition, the study was developed based on the Consolidated Criteria for Reporting Qualitative Research checklist [37].

## Results

This study was qualitative research that aimed to investigate facilitator and hinders factors for a successful retirement. A total of 22 in-depth interviews were performed with the interviewees. The mean age of retired participants was 63.15±6.30 years. Additionally, 63% of retired participants had a part-time occupation (Table 1). The mean age of about-to-retirement participants was 53.75±2.63 years. Concerning work experience, the mean work experience of about-to-retirement participants was 29 years. The education level of retired and about-to-retirement participants ranged from a diploma to a master´s degree. For retirement experts, work experience ranged from 8 to 20 years, and their education level ranged from a diploma to PhD in the field of research or executive. The results are summarized into 2 themes, 7 categories, and 20 subcategories (Annex 1).

### Barriers to a successful retirement

Barriers to a successful retirement were categorized into four groups, namely worsened health status, lack of a plan and retirement crisis, socioeconomic problems, and Inadequate supportive systems. Eleven subcategories were also developed, namely physical problems, psychological problems, Work holism and job burnout, lack of a plan, the transition from employment to unemployment, discrimination and social exclusion, Livelihood concerns, financial problems, and negative consequences of economic problems.

### Worsened health status

The elder participants reported physical and psychological problems, work dependency, and burnout as the major problems of a successful retirement. In combination with increased age, retirement results in decreased physical strength, leading to motor limitations, particularly due to diseases that have been ignored before retirement. The elderly feel more bored and lethargic after retirement and suffer from psychological problems, such as fear, stress, depression, and mood changes. Work holism and job dependency increase the difficulty of retirement adaptation, as the march to retirement is accompanied by work obsession and habits. After the transition to retirement, the elders feel decreased dignity and respect. These issues and stresses translate into excuse-making behaviours, inappropriate behavioural symptoms, and exacerbated physical and psychological pain. Even some cases might wish to die.

### Lack of a plan and retirement crisis

The transition from employment to unemployment, insufficient skills, and Lack of a plan for retirement is among the major barriers to a successful retirement. Unemployment is the major pitfall of a successful retirement; accordingly, sudden and unperceived unemployment results in the unpreparedness of the elderly, either mentally or purposefully (having a plan). Poor leisure skills (i.e., knowledge obsolescence) can be contributed to unsuccessful retirement. Therefore, they feel confused and aimless. Some retirees make extensive efforts to find alternative activities; however, insufficiency or lack of communication and skills results in failure or a trial-and-error cycle. Rather than a gradual problem, sudden retirement causes an unpleasant effect on retirement adaptation.

### Socioeconomic problems

Discrimination and social exclusion, livelihood concerns, financial problems, and negative consequences of macroeconomic problems are barriers to a successful retirement. Increased living costs, in combination with high medical expenditures related to older ages and chronic diseases, along with emotional deprivation due to social exclusion, create a profound and synergistic effect on retirees. Inappropriate perspectives, negative stereotypes, and incorrect beliefs and behaviours of family and society toward retirees cause self-perceived uselessness among the elderly; as a result, they feel dependency and gradual exclusion. Reduced wages and inadequate salary and income due to retirement result in financial imbalance and poor economic conditions. Therefore, most retirees try to find alternative income sources. Furthermore, retirement is accompanied by reduced social communication. Staying at home and its consequent isolation translates into negative effects on the elderly. The inflation created a heavy burden on the elderly, and they struggle with livelihood concerns, which in some cases have resulted in poverty.

### Inadequate supportive systems

This category includes a lack of appropriate plans and social services for the elderly and policies that are not appropriate for retirement. Retirement desires special attention. Deprivation of the elderly from some facilities due to retirement, lack of institutions to develop appropriate plans for the elderly, and inappropriateness of current services enhance the importance of addressing the priorities of this community. It seems that institutions responsible for retirement policies do not have a specific plan to facilitate retirement adaptation, and the few existing programs are unrealistic and do not have a specific application in the daily life of retirees. It can be attributed to a lack of applicable social policies, systemic problems, inefficient structures and bureaucracy, unawareness about retirees´ needs due to budget inefficiency, and a lack of reliable studies, statistics, and appropriate infrastructure. Retirees complained of inadequacies, lack of support, and not receiving desirable public welfare services. They were also dissatisfied with income discrimination between retirees and employees and political manoeuvres to ingratiate retirees and expressed their expectations to address their problems.

### Factors facilitating successful retirement

Factors that facilitate a successful retirement were categorized into three groups, including benefiting from social support, personal characteristics, and social participation. The subcategories were also categorized into nine groups, namely family support, the existence of social networks and appropriate relations, the positive impact of retirement associations, accepting retirement, mindfulness, self-efficacy, healthy lifestyle, humanitarian activities, and religious activities.

### Benefiting from social support systems

Family support, the existence of social networks and appropriate relations, and the positive impact of retirement associations were major facilitators of a successful retirement. Successful retirees are accepted and perceived by their family members and receive positive feedback, companionship, and empathy. Such families make work divisions and encourage retirees to perform various activities. By respecting retirees, family members use their experiences and guides. The supportive network understands retirees and tries to enhance their interactions and communications to establish strong social communications. In this situation, retirees benefit from a higher level of mental health, feel self-esteem and life satisfaction, and do not feel lost or lonely. They receive various forms of psychological and social support from family, peers, and the community.

### Personal characteristics

The acceptance of retirement, mindfulness, a positive view toward retirement, self-efficacy and a healthy lifestyle are important factors that contribute to adaptation to a successful retirement. By accepting retirement as a part of life, retirees adapt themselves. They develop rational plans for their leisure time using mindfulness and realistic expectations. They study and think. In addition, they have positive beliefs and values in life and strive for further satisfaction. A successful retirement is accompanied by feelings of the capability of performing daily activities, usefulness, and self-efficacy. They care about psychological and social health. They try to give meaning to their retirement life and follow a healthy lifestyle. By fully understanding their physical status and emotional gaps, retirees with a successful retirement try to handle these issues. They avoid tensions and life uniformity. Therefore, they feel happiness and calmness because they know retirement is not equal to a disability, and they seek to increase their quality of life using their abilities, efforts, and benchmarking successful cases.

### Social participation

The engagement of retirees in their communities through humanitarian, religious, and part-time activities is among the facilitators of a successful retirement. Due to not having a permanent occupation, retirees have more opportunities for social activities, including humanitarian and religious activities. This opportunity and importance of social participation fade the feeling of confusion and worthlessness and result in a feeling of freedom. They seize this opportunity for sharing experiences. These activities provide an opportunity to play a role and even earn money, leading to filling the free time and improving mental health.

## Discussion

This study intended to identify factors that facilitate and hinder a successful retirement. Retirement is a major life transition, particularly for the elderly, which was described as marriage or even re-born in this study. Adaptation to retirement is the process of getting along with changed living conditions caused by the end of a career life. If this process, along with preparation, gets shorter, it will positively impact the quality of life during retirement and lead to satisfaction. However, this “transition contains important adaptation issues with losing the occupational role and change in lifestyle” [7].

The findings showed the negative impact of changed physical and mental health on a successful retirement. The literature considers retirement a stressful event with psychosocial changes [38-40]. Although some studies reported that retirement is not necessarily associated with stress and psychological problems [41], most studies emphasized challenges related to retirement and its negative effects on psychological, social, and emotional well-being, depression, anxiety and stress, health status, isolation, and communications [22,25,42,43]. Early retirement also negatively affects physical health [44], and retirees, even in early retired cases, experience physical and psychological problems over time. Therefore, maintaining the physical and mental health of retirees has an important impact on their well-being, contributing to successful retirement [30]. For instance, Barbosa *et al*. reported the impact of physical health on adaptation to retirement [45].

The findings indicated the weaknesses and problems of supportive systems, including inappropriate retirement policies, insufficient skills, and lack of planning, as barriers to a successful retirement [46]. Also, the inadequate supportive systems category is mostly cited by participants as a barrier to successful retirement (68 percent). It seems that the government should run campaigns to promote retirement preparation in the country, as planning and providing resources allow retirees to experience a smooth adaptation to this important life event. Eagers *et al*. mentioned three stages for retirement, in which retirement preparation occurs before retirement (i.e., during the career) [18]. Previous studies showed that planning before retirement increases welfare and well-being after retirement. In addition, planning for retirement can modulate disorders caused by the lack of social networks [30,47], leading to a successful adaptation [48].

Economic problems were identified as a barrier to successful adaptation to retirement in this study, which is consistent with the results of previous studies that reported income adequacy during retirement as a predictor of a positive perspective toward retirement adaptation and satisfaction [33,49-51]. Economic changes are among the consequences of retirement [38]. Tavernier and Aartsen reported economic concerns and costs of living as important sources of stress during retirement and considered financial problems a factor that reduces the social participation of the elderly [52]. The insufficiency of financial resources causes negative and restrictive feelings in retirees and can affect the meaning of life for them. In addition, it might cause the limitation of resources and leisure time, leading to loneliness and a lack of opportunities, which in turn negatively affects the health of retirees [53]. Seventy-seven percent of participants in this study cited social support systems as a key facilitator to a successful retirement. The findings also showed an association between benefiting from social support, including support of friends and family members, having an appropriate social network, and maintaining social communications, with adaptation to a successful retirement. In the same vein, the literature supports the impact of social sources, including family and friends support, as the most important support source. In addition, there is an association between social interaction with the well-being of retirees and giving meaning to retirement [30,53]. Benefiting from emotional sources is associated with higher satisfaction from retirement. Moreover, declined social network after retirement causes dissatisfaction with life and poor health [30]. While emphasizing the importance of social interactions for retirement [25], previous studies reported the disruptive effect of this stage on relationships and losing friends and work-related relationships, leading to loneliness [3] and reduced life expectancy [19].

According to role theory [7], while emphasizing the effect of leaving a role on personal and interpersonal characteristics during retirement, the consequences, such as reduced well-being, satisfaction with life, self-worth, self-image [43], and ultimately anxiety and depression, are reported for this period [7]. Previous studies reported the importance of social relations during retirement [25] because retirement is linked with loneliness [54], and in the absence of support, it can lead to incompatibility with retirement.

Personal characteristics were identified as another contributor to a successful retirement. Previous studies also reported personal and motivational sources as characteristics that facilitate adaptation to retirement [47,55,56]. Positive personal sources give retirees a greater sense of control and dominance to address retirement challenges [57]. The findings showed that a positive perspective might result in a successful retirement. Consistent with the findings of previous studies, a positive perspective plays a key role in adaptation and satisfaction with life because, apart from giving meaning to life, it can protect the individual against undesirable conditions, such as illness. A positive attitude results in seizing the opportunity provided by issues that deem negative to learn or change [53]. Therefore, positive psychological factors contribute to increased preparedness for retirement and successful retirement life [58].

The findings showed that successful retirees do not consider retirement a problem but rather an opportunity to shape a new lifestyle. Having a healthy plan and lifestyle were among the factors that contribute to a successful retirement. According to the literature, a healthy lifestyle, including physical exercise, is associated with the well-being of retirees [30]. Successful retirees can do various activities, including housekeeping, gardening, and/or temporary occupations [53]. The findings indicated the impact of social participation on facilitating a successful retirement. In the same vein, some studies reported that participation in social services improves the well-being of retirees [30]. Previous studies demonstrated that retirement is associated with opportunities for social participation due to reduced occupational responsibilities and increased free time. In addition, the continuation of satisfactory and professional activities, including part-time and volunteer-based activities, affect health and life satisfaction after retirement [59], and volunteer-based activities create a meaningful sense of retirement life [60]. Previous studies showed that successful retirees define and maintain a specific daily routine by participating in various activities [53].

Therefore, according to the theory of activity by Atchley (1999) and in the light of activity theory, Bernice Neugarten (1964) asserted replacing job roles with recreational or voluntary activities is proposed for retirees to prevent the negative effects of retirement crisis on self-concept and continuation of the middle-aged lifestyle [7,61]. Retirement brings comfort and freedom from pressures caused by occupational responsibilities, translating into opportunities for growth and greater participation in family and social roles [7,59]. In addition, the continuation of satisfactory activities and roles that are based on the retirees´ skills, either part-time or voluntary, enhances both health status and satisfaction with life [59]. In a study conducted on retirees, Kujala and Moen showed that participants did not accept the retirement concept as a period characterized by leisure and occupation abandonment. They reported voluntary activities as a source of creating meaning [60].

**Study limitations:** although the qualitative design helps achieve a deep understanding of retirees´ experiences, caution should be taken when generalizing the results of teachers (participants with high education levels) to lower-educated workers. It seems that future studies should investigate factors that contribute to retirement following a retrospective longitudinal or prospective cohort design.

## Conclusion

This study considered retirement a phenomenon with several psychosocial challenges and an opportunity to grow, which various factors either facilitate or hinder it. Considering the consequences of retirement and focusing on the factors that facilitate or hinder a successful retirement in the expansion of training, interventions, and programs intended to prepare individuals for retirement can improve the current status. During the retirement process, individuals try to adapt themselves to retirement from various aspects, such as financial, social, and emotional. A successful retirement is a personal, family, and social phenomenon, and everyone plays a role in the achievement of a successful retirement. Retirement is associated with a changed identity. In the absence of gradual adaption using personal abilities and family and social support, the transition to retirement will result in losing identity and might affect the achievement of a healthy society. Therefore, retirement programs should consider strengthening psychological sources and social support for the elderly, as these factors facilitate the achievement of a successful retirement. **Recommendations:** periodic preventive interventions help retirees better adapt to retirement. As retirement is accompanied by reduced income, particularly for the elderly, retirees are faced with several economic problems that might affect their quality of life. Traditionally, retirement arrangements focus on financial and medical benefits and ignore social and psychological issues. In strategies and plans, politicians can tackle social inequalities by addressing factors that hinder and facilitate people's transition to retirement. Modifying existing retirement policies and drafting legislation for older workers who want to stay in the labour market increases their chances of employment. Also, retirees can apply their job experience in their field of work as a consultant. We don't have a comprehensive retirement planning program for our employees about to retire. We suggest focusing more on planning for retirement Using consultation and appropriate training to help retirees and their families navigate retirement and manage the economic aspects of life will facilitate a smooth and safe transition. Providing the opportunity to pursue interests and hobbies, learning how to engage in leisure activities, and continuing social relationships are some of the priorities in achieving a successful retirement. Future interventions should strengthen the cognitive skills of retirees, including the effect of knowledge of developmental changes on cognitive function, emotional experiences, and pursuit of goals, to prepare them to better cope with challenges related to this important event.

### What is known about this topic

- *Many reports estimate that the retiree population is growing, and this phenomenon presents complex challenges and opportunities for retirees;*
- *Retirement is a significant event in life and implies changes in various aspects that require more consideration specifically in the psycho-social aspects;*
- *Determining the factors that impact retirement helps maintain and improve health and quality of life and leads to a successful retirement.*

### What this study adds

- *To our knowledge, this is the first qualitative study to explore the facilitators and barriers based on Iranian participants' views of successful retirement;*
- *Individual characteristics, the meso, and macro factors such as psychological, social, and economic before the transition to retirement can support or prevent successful retirement, and social support systems play a significant role in successful retirement and lead to more satisfying retirement life;*
- *Inadequate support at the macro-level can influence as a barrier to successful retirement, so policymakers and health care providers should consider this to develop effective retirement strategies and interventions.*

## Annexe

**Annex 1**: categories and subcategories related to successful retirement (PDF 218 KB)
